# Human Activity Recognition: A Comparative Study to Assess the Contribution Level of Accelerometer, ECG, and PPG Signals

**DOI:** 10.3390/s21216997

**Published:** 2021-10-21

**Authors:** Mahsa Sadat Afzali Arani, Diego Elias Costa, Emad Shihab

**Affiliations:** Department of Computer Science and Software Engineering, Concordia University, Montreal, QC H3G 1M8, Canada; diego.costa@concordia.ca (D.E.C.); emad.shihab@concordia.ca (E.S.)

**Keywords:** human activity recognition (HAR), early fusion, 3D-accelerometer (3D-ACC), electrocardiogram (ECG), photoplethysmogram (PPG)

## Abstract

Inertial sensors are widely used in the field of human activity recognition (HAR), since this source of information is the most informative time series among non-visual datasets. HAR researchers are actively exploring other approaches and different sources of signals to improve the performance of HAR systems. In this study, we investigate the impact of combining bio-signals with a dataset acquired from inertial sensors on recognizing human daily activities. To achieve this aim, we used the PPG-DaLiA dataset consisting of 3D-accelerometer (3D-ACC), electrocardiogram (ECG), photoplethysmogram (PPG) signals acquired from 15 individuals while performing daily activities. We extracted hand-crafted time and frequency domain features, then, we applied a correlation-based feature selection approach to reduce the feature-set dimensionality. After introducing early fusion scenarios, we trained and tested random forest models with subject-dependent and subject-independent setups. Our results indicate that combining features extracted from the 3D-ACC signal with the ECG signal improves the classifier’s performance F1-scores by 2.72% and 3.00% (from 94.07% to 96.80%, and 83.16% to 86.17%) for subject-dependent and subject-independent approaches, respectively.

## 1. Introduction

With the recent increase in the use of smart phones and wearable devices, we can record and access a plethora of raw data and information from built-in inexpensive sensors. Human activity recognition refers to analyzing these data to extract meaningful information about human daily habits and physical activity patterns [1]. Breakthroughs in HAR research have led to various applications in health care and rehabilitation, elderly fall detection, fitness trackers, assisted living and smart homes [2]. One of the most frequently used sources of data for activity recognition purposes is the inertial sensor [3,4,5]. Inertial measurement unit (IMU) contains a triaxial accelerometer (3D-ACC), gyroscope, and magnetometer to measure velocity and acceleration, rotation, and strength of a magnetic field, respectively. Based on previous studies, the 3D-ACC sensor outperforms gyroscope and magnetometer, however, combining 3D-ACC with gyroscope yields better performance in classifying activities [6]. This suggests sensor combination has the potential to offer better classification power for a HAR system.

HAR research leverages multiple approaches to advance the accuracy and performance of systems, such as different sensor positioning [1], varying feature extraction approaches [7] and exploring several classification methods [8,9] to extract more informative knowledge from a dataset and enhance the HAR system performance. Bayat et al. evaluated the effectiveness of different 3D-ACC sensor placements in recognizing human activities [10]. Participants were instructed to perform tasks first using the smartphone in their hands, then perform the same tasks with the phone in their pockets. Based on the reported result, smart phone positioning in the hand and in the pocket yield similar results in the HAR models. Casale et al. used data acquired from only one 3D-ACC sensor and proposed a new set of feature extraction methods to be used with a random forest classifier in recognizing five different daily activities [11]. They observed that the new feature set is more informative compared to the commonly used feature sets; thus, they reported model performance enhancements after using the new feature set. In many cases, researchers evaluate the significance of one signal individually or IMU signals in combination, thus, the combined contribution of other types of signals (e.g., bio-signals) requires more exploration [12,13].

To overcome the limitations of using a single sensor, researchers have explored the idea of applying fusion methods to enhance a HAR system [4]. Fusion methods refer to any integration of information from different sources, at the sensor, feature or classifier levels [4]; thus, they can be categorized in early fusion and late fusion methods [14]. To elaborate, early fusion refers to combining raw data or extracted features, and then feed the new combined dataset or feature set to a classifier [15]. It is relevant to mention that combining features extracted with different approaches, i.e., time domain and frequency domain features, is also considered as early fusion [16]. Another method of combining for a HAR system is the late fusion, or decision-level fusion. As the name implies, in this method, the models are trained separately on each signal, but their predictions are combined into an ensemble model (e.g., voting system), that predicts the final classifications [14]. In this study, we apply the early fusion method, thus, in the following, we elaborate more on previous works in which researchers applied the early fusion method.

### 1.1. Early Fusion with IMU

Early fusion methods fuse extracted features from different signal sources into a combined dataset, which serves as input for a Human-Activity classifier. Several works have applied this technique to improve the performance of classifier models. Chung et al. applied the sensor fusion approach by placing eight IMU sensors on different body parts of five right-handed individuals [1]. They trained a Long Short-Term Memory (LSTM) network model to classify nine activities. Based on their results, to get a reasonable classification performance, one sensor should be placed on the upper half of the body and one on the lower half; particularly, on the right wrist and right ankle. Regarding signal fusion, the authors stated that a 3D-ACC sensor combined with a gyroscope performed better (with accuracy 93.07%) than its combination with a magnetometer. In another study, Shoaib et al. followed the same data-level fusion approach and generated their own dataset by placing one smart phone in a subject’s pocket and another one on his dominant wrist and recording 3D-ACC, gyroscope and linear acceleration signals [13]. The authors tried different scenarios, such as combination of 3D-ACC and gyroscope signals, which they claim leads to more accurate results, particularly for “stairs” and “walking” activities. Moreover, they claim that the combination of signals captured from both the pocket and wrist improves the performance, especially for complex activities.

### 1.2. Early Fusion with Bio-Signals

Bio-signal refers to any signal generated by a living creature that can be recorded continuously [17]. Given that the heart rate is sensitive to physically demanding activities [18], can we rely on bio-signals to complement 3D-ACC sensors in recognizing certain types of activities? Bio-signals sensors have been shown to be quite accurate in capturing the bio-signals [19], but they have not yet been extensively explored in the context of HAR systems. Park et al. performed an experiment to extract Heart-Rate Variability (HRV) parameters from recorded electrocardiogram (ECG) data and combined it with a 3D-ACC signal for HAR researches [20]. They employed a feature-level fusion approach by fusing features extracted from HRV and 3D-ACC signals and classified five activities by examining three different scenarios. First, using only four features extracted from 3D-ACC (83.08%); second, considering 31 more features extracted from the ECG signal along with the ones used in the first scenario (94.81% ). Finally, using features extracted from 3D-ACC and only some selected features from ECG signal, this combination outperformed previous scenarios by achieving a 96.35% accuracy. Park et al. conclude that the ECG signal is a good complementary source of information along with 3D-ACC for HAR researches. Tapia et al. applied early fusion by recording an acceleration signal obtained from five 3D-ACC in addition to heart rate (HR) information [21]. The authors applied a C4.5 decision tree and Naïve Bayes classifier to classify 30 gymnastic activities with different levels of intensity. They claim that adding HR to 3D-ACC can improve the model performance by 1.20% and 2.10% for subject-dependent and subject-independent approaches, respectively. Based on [21], for the subject-independent approach, different fitness level and variation in heartbeat rate during non-resting activities are potential reasons for this minor recognition improvement.

As for photoplethysmogram (PPG) signal fusion, Biagetti et al. investigated the level of contribution of a PPG signal in addition to a 3D-ACC signal toward accurately detecting human activities [22]. The authors proposed a feature extraction technique based on singular value decomposition (SVD) in addition to Karhunen–Loeve transform (KLT) method for feature reduction. According to the authors, employing only a PPG signal is not enough for physical activity recognition. Thus, they compared applying only a 3D-ACC signal with a combination of PPG and 3D-ACC, consequently, they conclude that signal fusion incremented the overall accuracy by 12.30% to 78.00%. In another study, Mehrange et al. used a single PPG-ACC wrist-worn sensor placed on the dominant wrist of 25 male subjects to evaluate fused HAR system power in classifying indoor activities with different intensity [23]. They extracted time and frequency domain features and fed them to a random forest classifier. In terms of contribution level of PPG-based HR-related features in classifying activities, their results suggest a very slight overall improvement. Regarding the activity performance, HR addition did not help the classifier to indicate most of the activities except for intensive stationary cycling with 7% improvement in accuracy.

### 1.3. Our Contribution

As summarized above, studies have shown that the combination of one type of bio-signal with 3D-ACC enhanced the HAR system’s performance. Our study differs and complements the former studies in the following ways. First, thanks to the dataset that we used, we have 3D-ACC, ECG and PPG signals all recorded simultaneously and related to same group of subjects, thus, beside evaluating the added value of bio-signals to 3D-ACC, we can also compare the significance of each of the mentioned bio-signals. Moreover, we investigate the impact of bio-signal, not only on the overall performance of the HAR models, but also per single activity, to assess the impact of bio-signals on each set of activities.

To compare the performance of different signals, we analyze the data acquired from 3D-ACC, ECG and PPG sensors individually. Moreover, we use fusion methods to combine data from mentioned signals to examine their contribution level in the HAR system’s output. To analyze the signal’s contribution in HAR systems, we segment the signals, using a sliding window method to extract time and frequency domain features. Finally, we train random forest classifier models for subject-dependent and subject-independent setups. We evaluate the bio-signals significance in HAR using two types of models: subject-specific and cross-subject models. Both models are commonly used in HAR systems and research, and more importantly, each has its advantages and disadvantages [24,25]. Subject-specific models are personalized models, trained and evaluated using the data of a single user. Hence, subject-specific are usually more accurate than cross-subject models, at a cost of requiring training data from the target user.

A cross-subject model, on the other hand, is trained on multiple users and attempts to recognize the activity of a previously untrained user. This model tends to be more generic and is commonly used in practice, since cross-subject models are cheaper to train and easier to deploy [26,27].

Therefore, we formulate our research questions to cover both subject-specific and cross-subject models. Thus, in our study, we focus on answering two research questions. First, we investigate the contribution level of each source of 3D-ACC, ECG and PPG signals in subject-specific HAR systems (RQ1), and then, in cross-subject systems (RQ2). Moreover, we have the advantage of having the three signals from the same individuals performing the same activities. Hence, we can investigate: (1) the contribution level of the bio-signals when added to the 3D-ACC signal, (2) and compare the performance of ECG and PPG with each other. To summarize, we provide an overview of our contributions:

- To the best of our knowledge, this is the first study to compare the combined performance of the 3D-ACC, ECG and PPG signals recorded simultaneously from subjects performing the same set of activities. We use hand-crafted features to evaluate the performance of classifiers for HAR;
- We investigate the significance of bio-signals and compare the usefulness of ECG and PPG signals in HAR;
- We investigate the impact of combining a 3D-ACC signal with an ECG signal in recognizing some specific activities in detail. For instance, the importance of the ECG signal in distinguishing walking activity from ascending/descending stairs.

The rest of this article is organized as follows; in Section 2, we elaborate on the characteristics of the sensors and signals we evaluate and the used dataset. Next, in Section 3, we explain our methodology and workflow, from data pre-processing to feature extraction and selection. We allocate Section 4 to classification and evaluation methods. Section 5 describes the results and findings of our study. We discuss in details the impact of ECG signal in HAR system’s performance in Section 6. Finally, we conclude our study in Section 7.

## 2. Studied Dataset

In this section, we describe the data used in our study. First, we illustrate the characteristics of the signals under-study, in Section 2.1, which is relevant to our methodology. Then, we describe the dataset used in this study in Section 2.2.

### 2.1. Sensors and Signals

In our study, we consider three sources of signals, 3D-ACC, ECG and PPG, for each of which we outline a brief explanation. An IMU sensor is a set of measurement units placed together in one device to capture information about the kinetic status of a device. Usually, the IMU sensor includes an accelerometer, gyroscope and magnetometer sensors. However, the original dataset did not contain gyroscope and magnetometer data, thus, they were not included in our study. Instead, we focus on the 3D-ACC, which is a source of information frequently used in HAR research and applications. This sensor is an electromechanical device converting mechanical forces into electrical signals. Thus, 3D-ACC signals are capable of measuring constant forces caused by gravity and rotation along axes, in addition to dynamic forces such as acceleration and vibration [28]. Having this knowledge is critical for the feature extraction phase.

**Figure 1:**
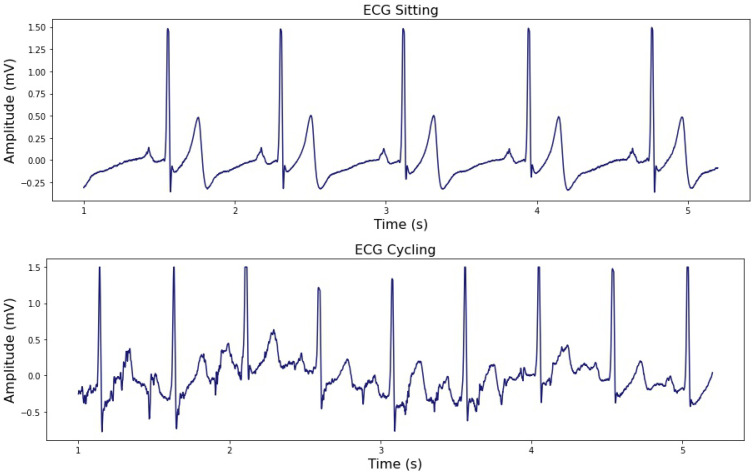
Five-second window of an ECG signal related to “Sitting” and “Cycling” activities performed by one subject.

Bio-signals, on the other hand, are capable of capturing meaningful information about the human body. ECG is one of the bio-signals and generated by the electrical activity of the heart. In order to record this electrical activity, a certain number of electrodes must be placed on a person’s chest; these electrodes record changes in voltage during each phase of the cardiac cycle, then, the recorded voltage is plotted against time based on the sampling rate frequency. The ECG signal has a specific pattern and a complete ECG period is made up of different intervals corresponding to a specific phase. The most well-known and obvious peak in one period of ECG signal is called the R peak which represents one heartbeat [29]. Counting R peaks in a fixed time interval is equivalent to the number of heartbeats during that specific time interval, thus, this information is capable of illustrating how fast the heart is beating, which may be a source of information in HAR research. In Figure 1, we compare two 5-s time windows of an ECG signal related to “sitting” and “cycling” activities.

Recently, another source of information has been used for HAR research called PPG signal. This signal is generated when infrared light passes through a human finger, wrist or earlobe, then captured by a light-detector after crossing the body part. During this process, some of the light is absorbed by the skin, bones and especially by the hemoglobin protein in the red blood cells, the rest of the light is recorded. The resulting signal is called the PPG signal [30]. Every time the heart pumps blood throughout the body, more lights gets absorbed by the hemoglobin protein and the PPG signal can represent this as heart beats. An advantage of using a PPG signal over ECG is that PPG can be recorded via a wrist-worn device, which is a more convenient solution for a subject to wear, than ECG signals that need a chest-worn device. However, PPG signals suffer from motion artifacts. Motion artifacts refer to any sort of voluntary or involuntary movements that are recorded by the PPG sensor but are actually noise associated with the signal. Luckily, there are ways to eliminate motion artifacts or even use them; as Boukhechba et al. analyzed the PPG signal and decomposed this signal to cardiac and respiratory signals; moreover, they took advantage of motion artifact noise associated with the PPG signal to recognize five types of human daily activities [31].

### 2.2. Dataset

We use the PPG-DaLiA dataset in our study [32]. This dataset was collected by Reiss et al., in which 15 volunteers, within the age of 21–55 years took part. Reiss et al. used two different devices to acquire the desired signals. A chest-worn device, RespiBAN [33] was placed on each subjects’ chest to record ECG signals, their respiration, and 3D-ACC at a 700-Hz sampling rate. In addition, the subjects wore a device on their non-dominant wrist called Empatica E4 [34] to collect 3D-ACC at a 32-Hz sampling rate, blood volume pulse (BVP) signal which contains the PPG signal at 64 Hz, electrodermal activity (EDA) and body temperature both at 4 Hz. After placing the mentioned devices on the volunteers’ chest and wrist, Reiss et al. asked them to perform some daily activities: sitting, ascending and descending stairs, playing table soccer, outdoor cycling, driving a car, being on lunch break, walking and working. Beside the mentioned activities, the authors also recorded the transient activities in between each of the aforementioned activities.

A noteworthy point about the PPG-DaLiA dataset is that the initial motivation for its collection was not for HAR research, but for extracting heart rate estimations using the PPG signal. This is why the chest-worn device sampling rate is relatively high compared to wrist-worn recorded signals in this dataset. We select this dataset for the following reasons; first and foremost, the experiments were performed indoors and outdoors which makes it more realistic and closer to daily life experiences compared to in-lab signal recording. While the researchers provided some protocols to instruct participants, they were free to execute each task in their own natural way. In addition to the recorded dataset, labels were provided for each activity, along with other significant information about each individual’s age, height, weight, fitness level, gender and skin type. Each subject performed all the activities for a total duration of 2.5 h. Table 1 provides detailed information about each activity completion protocol.

We analyze five human activities from the dataset: sitting, ascending/descending stairs, playing table soccer, outdoor cycling, and walking. It is important to highlight that this dataset is an imbalanced dataset. That is, more than 50% of all the instances are related to walking and sitting activities, 27% and 24%, respectively; and the smallest category is playing table soccer which makes up 13% of the entire dataset.

We disregard the remaining recorded activities, such as driving a car, lunch break, and working, because these activities are categorized as sequential, concurrent or interleaved human activities, and analyzing them is beyond the scope of this experiment [35]. Among all the recorded signals, we only consider the wrist-worn 3D-ACC, PPG and chest-worn ECG signals for our study. We disregard the chest-worn 3D-ACC data, as we already gathered 3D-ACC data from the wrist device, which provides better quality data for a HAR system [1,13]. Finally, we also disregard the data related to one of the subjects due to hardware issues during data recording [32].

## 3. Feature Extraction and Selection

**Figure 2:**
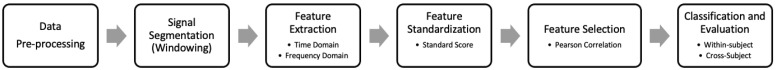
Human activity recognition workflow.

In this section, we describe the methodology used in our study to evaluate the importance of the three different signals in HAR. Figure 2 presents an overview of our methodology used to recognize human activity. We start by pre-processing the dataset to normalize data from different signals (and frequencies) and prepare the data for subsequent analysis. Next, we apply signal segmentation method, traditionally employed in HAR pipelines [36]. As our third step, we extract time and frequency domain features from each segment of the previous step. Then, we standardize the extracted features, so that all the features have zero mean and unit variance. Afterwards, we identify and remove highly correlated features and we train machine learning models with the remaining features. In the following, we explain the purpose and detailed procedure of each of the mentioned steps.

### 3.1. Data Pre-Processing

As described in Section 2, the dataset we use in this study contains signals with different sampling rates, as 3D-ACC was captured at 32 Hz while PPG was recorded at 64 Hz. To analyze the 3D-ACC and PPG signals captured from the wrist worn device, we up-sample the 3D-ACC signal from 32 to 64 samples per seconds. We decided not to down-sample the PPG signal, as this would mean eliminating half of the PPG dataset which was not appropriate. Thus, we up-sample the 3D-ACC signal by interpolating two-consecutive datapoints with their average value. We do not modify the original chest-worn ECG signal.

### 3.2. Windowing

Before we start extracting features from the data, we segment the entire signal into small sequences of fixed size (same number of datapoints). This approach is known as sliding window. The main intuition for the windowing technique is to retrieve meaningful information from the time series. Each single datapoint in the time series is not representative of any specific activity, however, a group of consecutive datapoints (a slice in the time series) is capable of providing insightful information about the human activity.

The size of the window is an important parameter in sliding window techniques. Each window must be wide enough to capture enough information for further signal processing and analyzing. However, the window size should not be too large, since larger windows may delay the real-time signal processing and the eventual activity recognition. The reason being that the model has to wait for the entire duration of the window to be able to start recognizing the next activity. Thus, there is a trade-off between capturing the appropriate amount of information and the speed of recognition. There is no standard fixed window size that researchers can utilize, as the appropriate window size depends highly on the characteristics of the signal. For instance, if we have a periodic signal, an adequate window size may be the one that is wide enough to cover at least one period of the signal in each segment.

Researchers have attempted different strategies to select an appropriate window size. One strategy is to use adaptive window sizes in which a feedback system is employed to calculate the likeliness of a signal belonging to an activity, then, the desired window size is selected based on the probabilities [37]. In most cases, however, researchers opt for fixed window sizes, Banos et. al. studied the impact of different window sizes on HAR accuracy [36]. They observed that many researchers have applied varying fixed window sizes from 0.1 to 12.8 s, although, nearly 50% of the considered studies have used 0.1 to 3 s window sizes.

**Figure 3:**
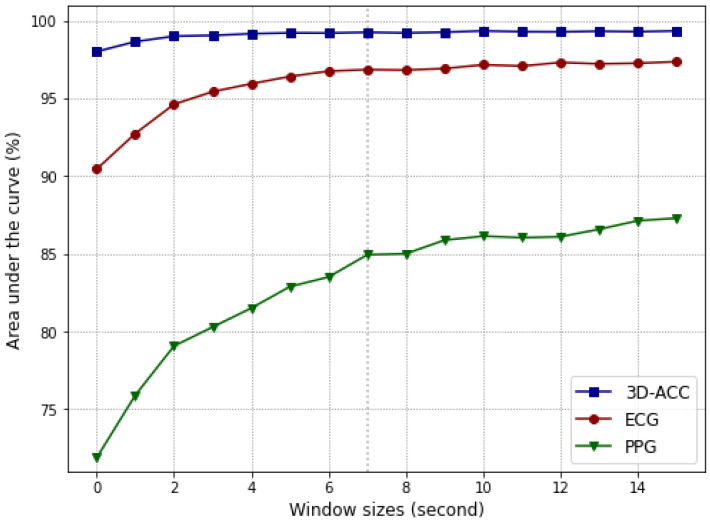
Comparison between different window sizes for 3D-ACC, PPG, ECG signals. X-axis: Window sizes represented in seconds. Y-axis: Area under the receiver operating characteristic curve after train and test random forest models.

In this study, we examine different fixed window sizes from 0.5 to 15 s on all three sources signals, and we select a window size of seven seconds. As depicted in Figure 3, larger window sizes provide only a slight improvement in the performance of the 3D-ACC signal. This means that smaller window sizes are still capable of capturing enough information out of the 3D-ACC signal and at the same time preserve a reasonable speed in recognizing an activity. Contrasting with 3D-ACC signals, larger window sizes are more informative for the bio-signals (ECG and PPG). Because having a larger window means capturing more than one period of cardiac activity in one window, thus, the heartbeat rate can also be taken into account.

Given that we aim to compare the performance of 3D-ACC and bio-signals, we must select equal window sizes, in terms of time duration, to have a reasonable comparison. Thus, we need to keep the balance between selecting smaller window sizes for 3D-ACC and larger ones for PPG and ECG. We opt to select a window size of seven seconds, which offered a good balance across all signals.

Since each window of the segmented signal is not completely independent and identical from its neighboring windows, we applied non-overlapping sliding windows. Based on the results obtained from Dehghani et. al., such signals are not independent and identically distributed (i.i.d.), so that overlapping would lead to classification model over-fitting [38].

### 3.3. Feature Extraction

After segmenting the signals in windows of seven seconds, we extract two types of features from each window: hand-crafted time and frequency domain features. In the following, we provide more detailed information about these two categories of features.

#### 3.3.1. Time-Domain Features

Time-domain features are the statistical measurements calculated and extracted from each window in a time series. As formerly described, we segmented five raw signals 3D-ACC, PPG and ECG with a sampling rate of 64, 64 and 700 Hz, respectively. In total, we extract seven statistical features from each of these windows. Table 2 presents the type of the features and their respective description. Features that we mention in the following table are easy to understand and are not computationally expensive, moreover, are capable of providing relevant information for HAR systems. Therefore, these features are frequently used in the field of HAR [13,39,40].

#### 3.3.2. Frequency-Domain Features

Transferring time-domain signals to the frequency domain provides insights from a new perspective of the signal. This approach is widely used in signal processing research as well as HAR field [39,40,41].

In the first step to extract frequency-domain features, we segment the raw time-domain signals into fixed window sizes. Then, we transfer each segmented signal into the frequency domain using the Fast Fourier Transform (FFT) method [42]. It is important to perform these two steps in the aforementioned order, otherwise, each window would not contain all the frequency information. That is, low-frequency information would appear in the early windows and, then, the high-frequency components would be placed in the last windows. By contrast, the correct way is that each window must have all the frequency components. After obtaining frequency components from each window, we extract eight statistical and frequency-related features. Table 3 presents different extracted features and a brief description for each of them.

**Figure 4:**
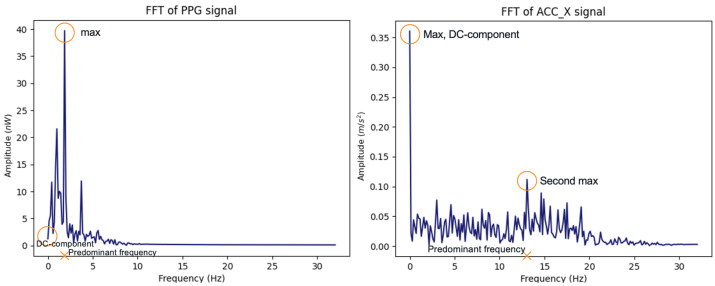
A seven-second window of PPG and X-axis of ACC signals transformed to frequency domain related to the “cycling” activity. Notice the difference between DC components, moreover, maximum amplitude and its corresponding frequency (predominant frequency) for different sources of signals.

From the frequency-domain features presented in Table 3, the DC component and dominant frequency are the less intuitive ones. Thus, next we explain these two features in more detail. The “DC component” is the frequency-domain amplitude value which occurs at zero frequency. In other words, DC component is the average value of signal in time-domain over one period. Regarding a 3D-ACC signal, its DC component corresponds to gravitational accelerations [43]. To be more precise, in the absence of device acceleration, the accelerometer output is equivalent to device rotation along axes [44]. This explains the reason why its DC-component value is relatively larger than the rest of the frequency coefficients in the same window [45]. As for bio-signals, however, the DC component is not highly greater than other frequency coefficients for which the reason is that bio-signals such as PPG and ECG are dynamic signals. Figure 4 represents PPG and X-axis of ACC signals related to a specific time span of seven seconds of cycling activity in the frequency domain. The difference between DC-component values between these two signals is clear.

Regarding a 3D-ACC signal, since the DC component is also the maximum amplitude, we decide to introduce another feature, namely, “second-max” to avoid feature redundancy and differentiate between mentioned features. However, for bio-signals, we only consider the maximum amplitude.

Dominant frequency is the frequency at which the highest amplitude occurs [46]. Based on this definition, again for a 3D-ACC signal, we disregard the amplitude corresponding to the zero frequency (DC component); instead, we consider the frequency corresponding to the second largest amplitude value (second max) as the “dominant frequency”. However, for the bio-signals, namely ECG and PPG signals, “dominant frequency” is based on the first maximum amplitude value.

### 3.4. Feature Standardization

Since we are examining different sources of signals with different characteristics, feature values will inevitably have different ranges, thus, we need to standardize the features before the model classification. For instance, the scale difference between X-axis of the ACC and the PPG signals have clearly distinct standard deviation of 0.18 and 64.2, respectively. This shows a huge difference in the amplitude of these signals which must be addressed. To standardize the extracted features, we calculate the standard score for each feature using Formula 1. This approach is used in some previous HAR studies [20,47].

(1) $$z_{i}=\frac{f_{i}-\mu }{\sigma },\;0\;\leq \;i\;<\;m$$

where for a given feature vector of size *m*, $f_{i}$ represents the *i*th element in the feature vector, μ and σ are the mean and standard deviation for the same vector, respectively. The resulting value, $z_{i}$, is the scaled version of the original feature value, $f_{i}$. Using this method, we reinforce each feature vector to have zero mean and unit variance. However, the mentioned transformation retains the original distribution of the feature vector. Note that we split the dataset into train and test set before the standardization step. It is necessary to standardize the train set and the test set separately; because we do not want the test set data to influence the μ and σ of the training set, which would create an undesired dependency between the sets [48].

### 3.5. Feature Selection

In total, we extract 77 features out of all sources of signals. Following the standardization phase, we remove the features which were not sufficiently informative. Omitting redundant features helps reducing the feature table dimensionality, hence, decreasing the computational complexity and training time. To perform feature selection, we apply the Correlation-based Feature Selection (CFS) method and calculate the pairwise Spearman rank correlation coefficient for all features [49]. Correlation coefficient has a value in the [−1,+1] interval, for which zero indicates having no correlation, +1 or −1 refer to a situation in which two features are strongly correlated in a direct and inverse manner, respectively. In this study, we set the correlation coefficient threshold to 0.85, moreover, among two recognized correlated features, we omit the one which was less correlated to the target vector. Finally, we select 45 features from all signals.

## 4. Classifier Models and Experiment Setup

In the following sections we explain the applied classifiers and detailed configuration for the preferred classifier. Next, we describe the model evaluation approaches, namely, subject-specific and cross-subject setups.

### 4.1. Classification

In our study, we examine three different machine learning models, namely, Multinomial Logistic Regression, K-Nearest Neighbors, and Random Forest. Based on our initial observations, the random forest classifier outperformed the other models in recognizing different activities. Thus, we conduct the rest of our experiment using only the random forest classifier.

Random Forest is an ensemble model consisting of a set of decision trees each of which votes for specific class, which in this case is the activity-ID [50]. Through the mean of predicted class probabilities across all decision trees, the Random Forest yields the final prediction of an instance. In this study, we set the total number of trees to 300, and to prevent the classifier from being overfitted, we assign a maximum depth of each of these trees to 25. One advantage about using random forest as a classifier is that this model provides extra information about feature importance, which is useful in recognizing the most essential features.

To evaluate the level of contribution for each of the 3D-ACC, ECG and PPG signals, we take advantage of the early fusion technique and introduce seven scenarios presented in Table 4. Subsequently, we feed the classifier with feature matrices constructed based on each of these scenarios. We use the Python Scikit-learn library for our implementation [51].

### 4.2. Performance Evaluation

In our study, we evaluate two types of models, the subject-specific model and the cross-subject model. In the following, we provide detailed explanation about these two models and evaluation techniques.

#### 4.2.1. Subject-Specific Model

Subject-specific models are the most accurate types of models, as they train and test using the data belonging to same user. Hence, it is important that we evaluate if bio-signals can be useful to make such models even better.

To evaluate the performance of our subject-specific model, we employ a *k*-fold cross-validation technique [52]. *K*-fold cross-validation is a widely-used method for performance evaluation and consists in randomly segmenting the dataset into *k* parts (folds). The machine learning model is trained on k−1 partitions and is tested on the remaining partition; this procedure repeats *k* times, always testing the model on a different fold. For each of the *k* runs, the evaluation procedure is done based on the scoring parameter. Finally, the average value of obtained scores is reported as the overall performance of the classifier.

As stated in Section 2.2, we have an imbalanced dataset, therefore, it is important to specify how to split the dataset into folds. We use the stratified *k*-fold method to preserve the proportion of each class label in each fold to be similar to the proportion of each class label in the entire set. Regarding scoring parameters, we evaluate our models with two metrics, namely, F1-score and area under the receiver operating characteristic (ROC) curve [53,54]. Since our study is a multi-class classification problem, we aggregate the mentioned scores using an average weighted by support.

In our case, to evaluate the subject-specific model, we consider one feature set related to only one subject and split it into a train set (80%) and a test set (20%). Subsequently, we apply the 10-fold CV technique on the training set and store the resulting F1-score and AUC measurements per fold. Finally, we apply the trained model on the test set, then, we record its classification performance in terms of F1-Score and AUC. Our objective of evaluating the model performance on the train set, and then on the test set, was to confirm that the model is not overfitting the data. An overfitted model fits perfectly on the train set, but has poor performance on the test set [55]. We repeat the described procedure 14 times, as many as the number of subjects. Eventually, we calculate the average F1-Score and AUC, over all subjects’ results and will report its performance in Section 5.1.

Subject-specific model is a subject-dependent technique, since we train the model on features related to one subject and then test the model using the remaining features belonging to the same subject; also known as “personal model” in the study of Weiss et al. [56].

#### 4.2.2. Cross-Subject Model

Cross-subject models are not as accurate as the subject-specific models [21], however, since such models are cheaper, in practice, these are more commonly used. Cross-subject models are cheaper because they do not require the user’s personal data, instead, are trained using data from other individuals. Therefore, knowing that bio-signals can contribute to this type of model is important to improve the generalization of the model.

To evaluate the performance of our cross-subject model, we use the Leave-One-Subject-Out (LOSO) evaluation method [57,58]. This method consists in training models on a group of subjects and testing the model on unseen individual data (test set). Similar to the *k*-fold, in a group with *n* subjects, the model is trained in n−1 subjects and tested on the remaining subject’s data. This process repeats *n* times, to cover all subjects in the test set, always using the other subjects’ data for training.

In our case, to evaluate the cross-specific model, we create a larger feature table consisting of 13 feature sets related to all subjects but one. We use this table as an input matrix to train the random forest model. Then, we test our model by the feature table of the remaining subject. Again, we aggregate F1-score and AUC measurement using average weighted by support. We rerun this process 14 times. Then, we calculate the average F1-Score and AUC over all subjects’ results and will report its performance in Section 5.2.

The aforementioned evaluation method, minimizes the risk of overfitting, moreover, it is subject-independent; and is also called “impersonal model” in the study of Wiess et al. [56]. Hence, if a classification model performs well given the LOSO evaluation method, then this model generalizes well to other subjects.

## 5. Results

In this section, we report the results obtained from aforementioned evaluation strategies. We answer our initial questions about how informative are each of the sources of signals and whether sensor fusion improves the performance of a HAR classifier. We organize the result section based on the scenarios presented in Table 4, that is, using only one source of signal (scenario 1, 2 and 3), considering a combination of two signals (scenario 4, 5, and 6), and scenario 7 which is related to 3D-ACC, ECG and PPG signals fusion. We conclude each subsection by reporting our observations regarding the activity performance of the HAR models.

### 5.1. RQ1: What Is the Contribution Level of Signals under Study in Subject-Specific HAR Systems?

**Figure 5:**
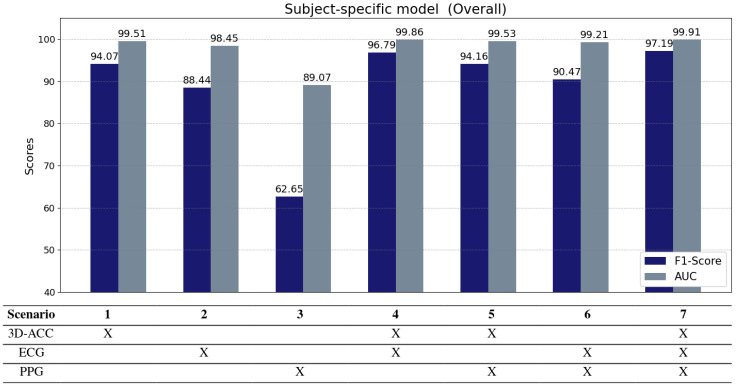
Subject-specific model results.

As stated in Section 4.2.1, we train and test subject-specific models and we are interested in the contribution level of each source of signals in these models. We report the results of the subject-specific model evaluation in Figure 5.

**One type of signal.** Considering only one source of signal, the 3D-ACC signal (Scenario 1) outperforms the other two bio-signals in recognizing human activities (Scenarios 1–3). Interestingly, a model using exclusively the ECG signal (Scenario 2) performs relatively satisfactory, with a comparable AUC performance to a 3D-ACC trained model, yielding a much better performance than the model trained solely with a PPG signal (Scenario 3).

**Two signals fusion.** When combining the 3D-ACC with the ECG signal (Scenario 4), the performance of the model surpasses the model using only 3D-ACC (Scenario 1). As shown in Figure 5, adding ECG to 3D-ACC improves the human activity recognition by 2.72% in terms of F1-Score. Our results suggest that including the ECG signal in HAR systems that are based solely on the 3D-ACC can slightly improve the model performance. However, adding a PPG signal to the 3D-ACC signal (Scenario 5) does not provide any significant enhancement for our HAR models. Combining solely bio-signals (PPG and ECG in Scenario 6) does not yield a model with superior performance compared to just a 3D-ACC model, even if they outperform models trained with one bio-signal (Scenarios 2 and 3).

**Three signals fusion.** Regarding Scenario 7, when we consider all three sources of signals, we realize that human activity recognition performance remains almost the same compared to Scenario 4 when we only took 3D-ACC and ECG signals into account. Therefore, we conclude that PPG signal fusion did not add any strength to the classifiers in our evaluation. In addition, it is obvious from Figure 5 that the PPG signal is not very informative, not exclusively nor in combination with other sources of signals for subject-dependent HAR systems.

**Figure 6:**
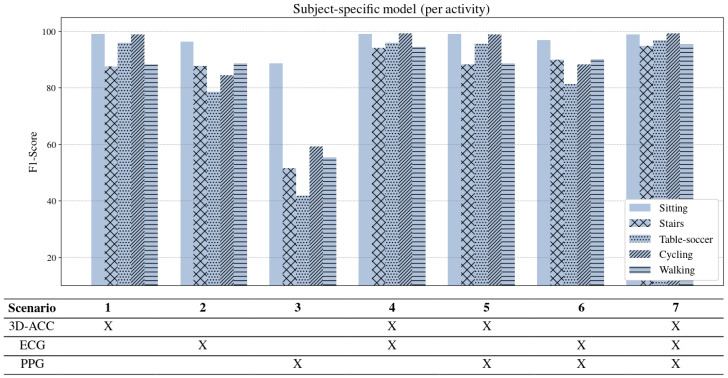
Subject-specific model results per activity.

**Per activity performance. **Figure 6 represents results of the subject-specific model per activity. It is noticeable that ascending/descending stairs and walking are the two activities that our models have difficulty distinguishing when using only the 3D-ACC signal. However, feeding the model with features extracted from both 3D-ACC and ECG signals (Scenario 4), improves the “stairs” and “walking” distinction significantly by 6.54% and 6.05% F1-score, respectively. An important takeaway from Figure 6 is that bio-signals have reliable power in distinguishing stationary activities such as sitting from non-stationary ones such as walking and cycling. When comparing the contribution of the PPG signal to the 3D-ACC per activity, we note that the combination did not yield any improvement in distinguishing the mentioned activities. In fact, the model performance when combining 3D-ACC and PPG is highly similar to its performance when we apply only a 3D-ACC signal (as in Scenario 1), which indicates an inadequacy of the PPG signal features in distinguishing any information not already captured by the 3D-ACC.

### 5.2. RQ2: What Is the Contribution Level of Signals under Study in Cross-Subject HAR Systems?

**Figure 7:**
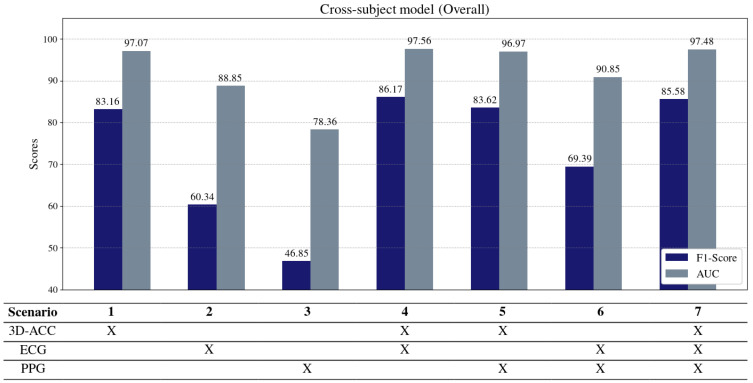
Cross-subject model results.

In Section 4.2.2, we mentioned the cross-subject models and the fact that these models tend to perform worse than subject-specific models, given that cross-subject are more general. Therefore, for the current evaluation setup, we observe a more significant contribution from bio-signals. Figure 7 shows the overall performance of the signals under study in terms of F1-score and AUC measurements (aggregated as stated above). As expected, the performance of cross-subject models has an overall lower performance than the subject-specific models. This decrease in performance is expected as cross-subject models are trained on other individual’s data, and individuals perform activities differently. Moreover, other factors, such as subject’s height, weights, gender and level of fitness may contribute in this variation.

**One type of signal.** According to Figure 7, the 3D-ACC signal provides the most informative data to our cross-subject models, yielding a performance with a F1-score of 83.16 (Scenario 1). Contrasting with the results observed for subject-specific models, a model trained only with ECG (Scenario 2) did not yield comparable performance with a 3D-ACC model (Scenario 1). Still, cross-subject models trained using only the ECG signal (Scenario 2) outperform the models trained exclusively with the PPG signal (Scenario 3), by 13.49% in terms of F1-score.

**Two signals fusion.** The combination of 3D-ACC and ECG signals (Scenario 4) has shown to improve the performance of our cross-subject model by 3% (F1-score), compared to using exclusively the 3D-ACC signal. Once again, a fusion of PPG and 3D-ACC signals has shown to not yield performance improvements (Scenario 5) to trained 3D-ACC models. Interestingly, combining both ECG and PPG (Scenario 6) yields a model with better performance than the models trained exclusively with ECG (Scenario 2) and PPG (Scenario 3), even if it still underperforms against the purely 3D-ACC trained model (Scenario 1). In the end, we conclude that the ECG signal can be complement well the 3D-ACC signal in HAR systems, while PPG did not provide informative data to our cross-subject models.

**Three signals fusion.** Once we fuse all three sources of signals, we observe a decrease in the model’s performance compared to just combining ACC-3D and ECG signals (Scenario 4). This further corroborates with the notion that adding the PPG signal to the combination of 3D-ACC and ECG signals is disadvantageous.

**Figure 8:**
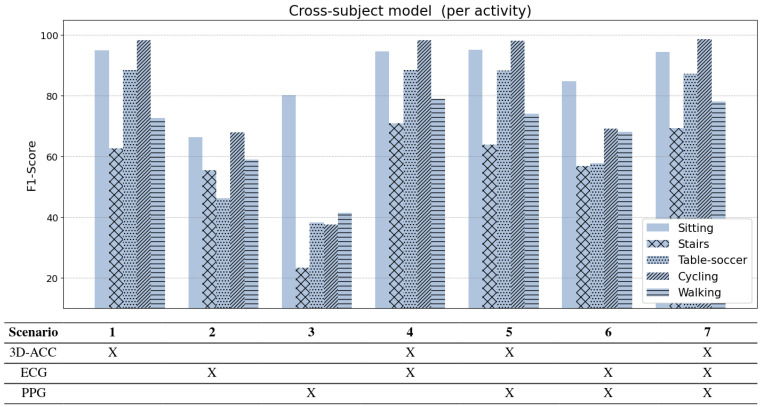
Cross-subject model results per activity.

**Per activity performance. **Figure 8 shows the performance of the models broken down per activity. First, we note that “cycling”, “table-soccer” and “sitting” activities remain rather stable in all models trained with 3D-ACC, exclusively or in combination. Second, cross-subject models often miss-classify “stairs” and “walk” activities. Once we fuse both the 3D-ACC and ECG signal, the model is better able to distinguish between the two activities, which explains the gain in the overall performance of the model. Third, models trained exclusively with bio-signals have very distinct performance profiles per activity. Note that PPG is reasonably good at distinguishing the “sitting” activity, as this is the least physically demanding activity in our dataset (lower heart rate). ECG models, on the other hand, outperform PPG models in all other activities, further corroborating that the ECG signal is, on average, more informative for cross-subject HAR models than the PPG signal.

## 6. Discussion

To elaborate more on the impact of the ECG signal in the performance of HAR models, we dive into the confusion matrices related to all subjects in both subject-specific and cross-subject models.

### 6.1. Subject-Specific

**Figure 9:**
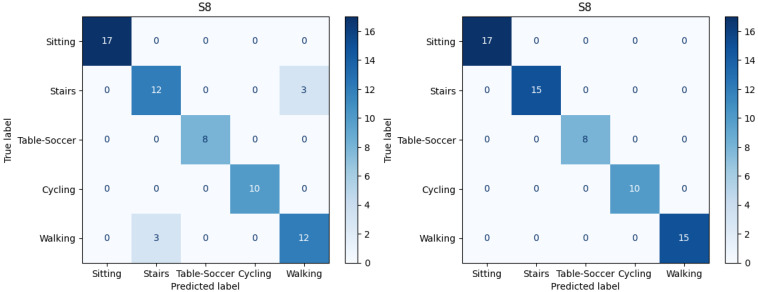
Comparison between confusion matrices in subject-specific models. On the **left**: the model performance when considering only 3D-ACC. On the **right**: the model performance when combining 3D-ACC with the ECG signal.

As stated earlier, considering only 3D-ACC signals, models already reach a high recognition performance of 94.07% F1-score. Thus, most of the instances in confusion matrices are labeled accurately. However, the activities of using “Stair” and “Walking” were frequently confused with each other. Therefore, we contrast the confusion matrices of the model which include only 3D-ACC (Scenario 1) against the models including both 3D-ACC and ECG signals (Scenario 4). We observe significant improvement in distinguishing the mentioned activities after adding the ECG signal. Figure 9 presents confusion matrices related to subject number 8 in the subject-specific model. On the left side of Figure 9, we can observe the model performance when considering only 3D-ACC. Note the instances which are miss-classified and confused between “Stairs” and “Walking” activities. On the right side of Figure 9, however, it is clear that after adding the ECG signal, the mentioned confusions are solved.

It is important to mention that for 10 out of 14 subjects we observe “Stairs-Walking” improvement after adding the ECG signal to 3D-ACC, however, in 3 out of 14 cases adding the ECG signal does not improve the “Stairs-Walking” classification. Moreover, in 1 case, the model perfectly distinguishes between “Stairs-Walking” by just using the 3D-ACC, leaving no space for improvement for the 3D-ACC and ECG fusion model.

### 6.2. Cross-Subject

Cross-subject models provide a more insightful analysis, since these models miss-classify activities more often, compared to subject-specific models. As depicted in Figure 7, using only the 3D-ACC signal, we obtained an F1-score of 83.16% which is relatively lower than the model performance in the subject-specific setup. After a detailed investigation in confusion matrices of the 3D-ACC trained model, we once again identify that the activities “stairs” and “walking” are miss-labeled. In addition to the mentioned pair of activities, another pair is miss classified in cross-subject models, namely, “sitting” and “playing table soccer”.

**Figure 10:**
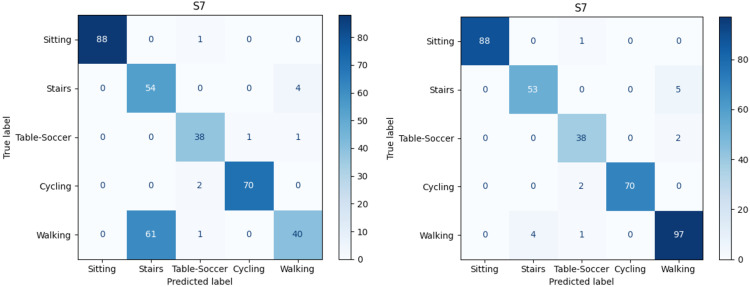
Comparison between confusion matrices in cross-subject models. On the **left**: the model performance when considering only 3D-ACC. On the **right**: the model performance when combining 3D-ACC with the ECG signal.

We once again compare the confusion matrices related models trained with 3D-ACC (Scenario 1) signal versus the model trained with both 3D-ACC and ECG signals (Scenario 4). We observe that the ECG signal significantly helps the model recognize “Stairs-Walking”, however, it does not add any value when it comes to distinguishing the “Sitting-Table-Soccer” pair. Figure 10 depicts both confusion matrices related to subject number 7 in the cross-subject model. The left side of Figure 10 is related to the model performance when considering only 3D-ACC; note the huge portion of “Walking” instances which are miss-classified as “Stairs”. However, on the right side of Figure 10, it is obvious that after adding the ECG signal, the “Stairs-Walking” detection enhances noticeably.

It is worth noting that for 9 out of 14 subjects, we observe “Stairs-Walking” improvement after adding the ECG signal to a pure 3D-ACC model. In 3 out of 14 cases, adding the ECG signal yielded no significant impact; and, in 2 out of 14 cases, the ECG signal addition resulted in a decline in the “Stairs-Walking” classification.

### 6.3. Feature Importance

We have shown that fusing 3D-ACC and ECG signals yielded the best performance in classifying human activities in our study. However, which features from both signals were the most relevant to our model? In this section, we present the feature importance ranking of the model that combines 3D-ACC and ECG (Scenario 4) using the cross-subject model, as we want to investigate the best features across multiple subjects. We calculate the feature importance using the Mean Decrease in Impurity (MDI) of our random forest model [59]. To aggregate the importance score for each model evaluated on a single subject, we calculate the average score for each feature over all the subjects and rank their importance score. As Table 5 shows, out of top 20 features, 16 features are related to the 3D-ACC signal and 4 of them to the ECG signal. Naturally, as 3D-ACC provides the best signal of the individual signal models (scenario 1), we expect to see a dominance of 3D-ACC features in the top-20 ranking. Interestingly, frequency-domain features rank higher than the time-domain ones, confirming that frequency-based features provide informative data to our classifiers. Another pattern that emerges is that, for 3D-ACC, the Y and Z axes are relatively more important than the X-axis of the accelerometer sensor. As the original authors of the PPG-DaLiA dataset collected the accelerometer using a wristband, when the subject’s hand is positioned alongside the body, the X-axis captures movement in the forwards-backwards direction, the Y-axis captures up-down movements, and finally, the Z-axis captures left-right movements [34].

### 6.4. Limitation and Future Work

As discussed in Section 2.2, a variety of recorded signals are provided in the PPG-DaLiA dataset, however, out of IMU sensors (3D-ACC, gyroscope, and magnetometer), the available signals are limited to the 3D-ACC. This limitation prevents us from further investigating the contribution level of bio-signals when combined with gyroscope, and magnetometer signals. Furthermore, our results show that there are varying levels of contribution of the ECG signal in scenario 4. This variation may be due to fitness level, thus, the heart activity, of an individual. Although the PPG-DaLiA dataset was provided along with the fitness level score for each subject, the majority of the subjects had a score of above 4 (the scores are in the range of 1 to 6 depending on how often an individual exercises), which makes it difficult to check the impact of fitness level on heart and, consequently, on fused HAR systems. In our future work, we plan to check the impact of other contributing factors, such as fitness level, height, weight, and age on the performance of a fused HAR system in cross-subject setup. Moreover, as stated earlier, there are two main fusion methods, namely, early and late fusion, we want to further evaluate the contribution level of the signals under study in the late fusion approach.

## 7. Conclusions

In this paper, we perform a comparative study to evaluate the contribution level of different sources of signals in HAR systems. We use a dataset consisting of 3D-ACC, PPG, and ECG signals recorded while 14 subjects were performing daily activities.

After examining different window sizes, we identify that window size of seven seconds is the best segment duration to obtain sufficient information out of the mentioned signals. Afterwards, we extract time and frequency domain features from each segment, then standardize features so that all the features have zero mean and unit variance. Next, we omit features with more than 85% correlation and select 45 features out of total 77 extracted features. We introduce seven scenarios of evaluation, including models trained solely by one source of signal and models trained by a combination of signal features. Finally, we evaluate two types of HAR models using Random Forest classifiers, a subject-specific model and a cross-subject model.

We conclude that in both subject-specific and cross-subject models, the 3D-ACC signal is the most informative signal if the HAR system design purpose is to record and use only one source of signal. However, our results suggest that the 3D-ACC and ECG signal combination improves recognizing activities such as walking and ascending/descending stairs. Moreover, we experimentally assess that features extracted from the PPG signal are not informative for HAR system, not exclusively, nor when applying signal fusion. Although, both bio-signals yield a satisfactory performance in distinguishing stationary activities (e.g., sitting) from non-stationary activities (e.g., walking, cycling). Overall, our results indicate that it might be beneficial to combine features from the ECG signal in scenarios in which pure 3D-ACC models struggle to distinguish between activities that have similar motion (walking vs. walking up/down the stairs) but differ significantly in their heart rate signature.

## Figures and Tables

**Table 1:** Type of activities and detailed protocol based on the study of Reiss et al. [32].

Activity	Protocol
Sitting	Sitting while reading
Ascending/descending stairs	Climbing six floors up and going down, repeated two times
Play table soccer	Playing table soccer, 1 vs. 1
Cycling	Cycling 2 km outdoors cycling with gravel and paved road condition
Driving a car	Driving on a defined road for 15 min
Lunch break	Includes queuing and fetching food, eating, and talking at the table
Walking	Walking back from the canteen to the office, with some detour
Working	Subjects’ work mainly consisted of working on a computer.
Transient periods	Each transition between activities

**Table 2:** Hand-crafted time-domain features and descriptions. Each of these features is calculated over datapoints within each window.

Hand-Crafted Time Domain Feature	Description
mean	average value of the datapoints
min	smallest value
max	largest value
median	the value at the 50% percentile
standard deviation	measures how scatter are the datapoints from the average value
zero-crossing rate	counts the number of times that the time series crosses the line y=0
mean-crossing rate	counts the number of times that the time series crosses the line y=mean

**Table 3:** Hand-crafted frequency-domain features and descriptions. Each of these features is calculated over frequency components within each window.

Hand-Crafted Frequency Domain Feature	Description
mean	average value of the datapoints inside one window
min	smallest value
max	largest value (only for bio-signals, PPG and ECG)
second-max	second largest value (only for 3D-ACC signal)
DC component	zero frequency component
standard deviation	measures how scatter are the datapoints from the average value
median	is the middle value after sorting datapoints from smallest value to the largest one
dominant frequency	is the frequency correspond to maximum energy (amplitude)
mean-crossing	counts the number of times that the time-series crosses the line y=mean

**Table 4:** Different proposed scenarios to evaluate the level of contribution for each of the 3D-ACC, ECG and PPG signals and their combination, in addition to the total number of applied time- and frequency-domains features after feature selection.

Scenario ID	Considered Signals	Total Number of Features
1	3D-ACC	24
2	ECG	12
3	PPG	9
4	3D-ACC + ECG	36
5	3D-ACC + PPG	33
6	ECG + PPG	21
7	3D-ACC + ECG + PPG	45

**Table 5:** Feature importance for 3D-ACC + ECG model (scenario 4) in the cross-subject setup.

Rank	Feature Domain	Signal	Feature Name	Importance Score
1	Frequency	ACC-Y	median	0.0990
2	Time	ACC-Z	median	0.0812
3	Frequency	ACC-Y	max	0.0799
4	Frequency	ACC-Z	standard deviation	0.0633
5	Frequency	ACC-Y	standard deviation	0.0626
6	Frequency	ACC-X	second-max	0.0579
7	Frequency	ACC-Y	mean-crossing	0.0535
8	Time	ACC-X	max	0.0475
9	Frequency	ACC-Z	mean-crossing	0.0454
10	Time	ACC-Y	max	0.0385
11	Time	ACC-X	median	0.0365
12	Time	ACC-Y	median	0.0361
13	Time	ACC-Y	mean	0.0343
14	Time	ECG	mean-crossing	0.0318
15	Time	ECG	zero-crossing	0.0279
16	Time	ECG	standard deviation	0.0276
17	Frequency	ACC-Z	predominant frequency	0.0275
18	Frequency	ECG	mean-crossing	0.0203
19	Time	ACC-Z	min	0.0191
20	Frequency	ACC-Y	predominant frequency	0.0188

## Abbreviations

The following abbreviations are used in this manuscript:

HAR	Human Activity Recognition
IMU	Inertial Measurement Unit
3D-ACC	Three dimensional accelerometer signal
ECG	Electrocardiogram signal
PPG	Photoplethysmogram signal
FFT	Fast Fourier Transform
CFS	Correlation based Feature Selection
AUC	Area Under Curve
ROC	Receiver Operating Characteristic
LOSO	Leave-One-Subject-Out cross validation
